# Short Italian Wilkins Rate of Reading Test for repeated-measures designs in optometry and neuropsychology

**DOI:** 10.3389/fpsyg.2024.1448817

**Published:** 2024-11-19

**Authors:** Maria De Luca, Davide Nardo, Giulia Carlotta Rizzo, Roberta Daini, Silvia Tavazzi, Fabrizio Zeri

**Affiliations:** ^1^IRCCS Fondazione Santa Lucia, Rome, Italy; ^2^Department of Education, University of Roma Tre, Rome, Italy; ^3^Department of Materials Science, University of Milano-Bicocca, Milan, Italy; ^4^COMiB Research Centre in Optics and Optometry, University of Milano-Bicocca, Milan, Italy; ^5^Department of Psychology, University of Milano-Bicocca, Milan, Italy

**Keywords:** Wilkins Rate of Reading Test, wpm, reading speed, repeated-measures, equivalent texts, Radner Reading Charts, practice effect, fatigue effect

## Abstract

**Background:**

The recently published New Italian version of the Wilkins Rate of Reading Test (standard Italian WRRT) was designed to measure reading speed in repeated-measures designs in research and/or clinical examinations. The test features 15 equivalent 10-line passages made up of unrelated words, adhering to the principles established by the Wilkins Rate of Reading Test in English (original WRRT).

**Aim:**

To develop a short Italian version of the WRRT (SI-WRRT), and to determine the equivalence across the new, shorter passages of text. The introduction of 5-line passages, instead of the original 10-line ones, aims to enhance the tool's suitability for the elderly or neuropsychological patients by reducing administration time.

**Method:**

The same 15 high-frequency Italian words from the standard Italian WRRT were used to generate 15 5-line passages for the SI-WRRT. Comprehensive eye examination and vision assessment, including the Radner Reading Charts, were performed before the administration of the SI-WRRT. Forty healthy Italian-speaking higher education students read the SI-WRRT passages aloud in random order. Reading speed and accuracy were measured offline from digital recordings of the readings. Equivalence across passages and the effects of practice and fatigue were assessed for reading speed and accuracy, along with test-retest reliability.

**Results:**

No significant difference in reading speed was found across 14 out of the 15 passages. In addition, no differences were observed in accuracy, and the error rate was very low. Practice and fatigue effects were minimal for reading speed, whereas they were absent for accuracy. Reading speed, the reference metric for the WRRT, showed moderate-to-good test-retest reliability.

**Conclusions:**

Equivalence was confirmed across 14 passages of the SI-WRRT. Therefore, the test may be suitable for examining the elderly or neuropsychological patients, as reading time of the 5-line passages is halved with respect to the standard Italian WRRT. However, the 5-line passages still allow the assessment of prolonged reading. Since one passage was not equivalent, we recommend avoiding the use of random rearrangements of words without formally checking their validity.

## 1 Introduction

In scientific research, there is often a need to measure the dependent variable more than once. In such situations, repeated-measures designs are commonly adopted, wherein the same participants are enrolled in experimental sessions in which a dependent variable is measured on multiple occasions over time (e.g., pre-, post-, and follow-up testing), or under different experimental conditions. For instance, repeated-measures designs are adopted when the same group of participants is exposed to different interventions or to an intervention protocol vs. a control condition. Whenever a measure is repeated, there is a potential issue with the equivalence of alternative test versions (e.g., Beglinger et al., 2005).

In the field of vision science, which encompasses disciplines ranging from optometry and ophthalmology to cognitive psychology and neuropsychology, repeated-measures designs based on multiple readings of texts (over time or across conditions) are typically applied to assess the efficacy of interventions for patients suffering from various vision deficits (e.g., Bailey and Lakshminaryanan, 1997), or reading interventions for patients with developmental (i.e., dyslexia; e.g., Tilanus et al., 2019; autism spectrum disorders; e.g., Ludlow et al., 2006) or acquired reading deficits (e.g., hemianopic alexia after stroke or traumatic brain injury; e.g., Spitzyna et al., 2007).

Irrespective of whether texts are used as diagnostic/monitoring tools in a clinical context, or as stimulus materials in experimental designs (e.g., Wilkins et al., 2005; Zeri et al., 2018), the same passage should never be used for multiple readings, to avoid practice/learning effects. Therefore, different passages need to be used, provided that they represent parallel forms (i.e., equivalent texts) that do not introduce factors that may interfere with the manipulated variable(s) and may hence produce unreliable (or hard-to-interpret) results. Different passages are equivalent when their basic characteristics (e.g., total number of words, syllables, and characters; number of words per line; number of lines of text) and text complexity (e.g., word frequency; syntax; sentence length; clause complexity; semantics) are comparable (e.g., Brussee et al., 2015; Radner et al., 2017; Trauzettel-Klosinski et al., 2012).

An alternative, effective way to generate homogeneous material for serial readings is to minimise the linguistic content of a text by using unrelated, high frequency words arranged in random order within a text line (e.g., Bailey and Lovie, 1980; Wilkins et al., 1996). This way, reading relies only on basic reading skills (as in primary school), without requiring any higher cognitive processing (e.g., inferring a meaning to generate predictions). Then, reading becomes dependent only on single-word processing and on visuoperceptual features of the stimulus, without any contextual influence (e.g., Stanovich et al., 1985). This has the further advantage of making the material suitable for testing children and adults with modest linguistic skills, as done in the Wilkins Rate of Reading Test (from here on, “original WRRT;” Wilkins et al., 1996), which uses passages made up of unrelated, short, high-frequency words (i.e., passages which are meaningless at the sentence-level).

The original WRRT was designed and adopted in optometry and vision science to assess visual performance in reading under different visual conditions (e.g., the use of different coloured overlays to aid reading difficulties; Wilkins et al., 1996). The test comprises 10 lines of text containing the same 15 words (repeated line by line), which are very common in the English lexicon, arranged in random order. The rationale of this test is to return reading speed as “words correctly read per minute” (wpm) using reading materials that neutralise/minimise the impact of syntax and semantics on the task. That is, the text content is as simple as possible, does not convey any meaning at the sentence-level, and is matched across conditions, so that any effect can be solely attributed to the experimental manipulation or clinical intervention at hand, and not to the text itself. Since Wilkins et al.'s aim was also to create materials that elicit visual stress, words within each line were closely spaced and line spacing was tighter. This way, reading is visually—but not cognitively—demanding, allowing the investigation of the effect of visuoperceptual factors and interventions on reading (Evans and Joseph, 2002; Monger et al., 2015; Northway, 2003; Wilkins, 2002). In addition, “single passage” versions in other languages were made available on Wilkins' website (http://www1.essex.ac.uk/psychology/overlays/rrt%20OC4.htm), including a 20-line Italian passage, although their validity was not determined. To create more passages, Wilkins et al. suggested generating equivalent forms of texts by randomly rearranging the words within each line. Therefore, multiple versions (up to four passages) of the original test were made available (Wilkins et al., 1996; Gilchrist et al., 2021). However, such passages were only assessed in terms of test-retest reliability (Gilchrist et al., 2021), rather than equivalence.

Conversely, we hypothesised that random rearrangements of high-frequency words *per se* may not necessarily return equivalent passages, as a given random order may accidentally form meaningful word sequences (which would otherwise be unrelated), possibly impacting on reading speed [e.g., “come see the play,” or “you see the dog” in Wilkins et al. (1996)]. Such concern prompted us to assess the equivalence across passages, which was formally tested and confirmed (alongside test-retest reliability) in a recent study introducing the New Italian version of the Wilkins Rate of Reading Test (from here on “standard Italian WRRT;” Zeri et al., 2023). In that study, we also increased the number of passages for protocols requiring more than 4 experimental conditions or repeated measures. Hence, the standard Italian WRRT features 15 equivalent passages. The structure and constraints of the original WRRT were maintained, and a transliteration instead of a direct translation was adopted. In the standard Italian WRRT, participants achieved an average reading speed of 167.3 wpm. A passage was read in <1 min (mean ± SD: 54.9 ± 0.6 s, computed across mean values of single passages; range across individuals: 38.0–79.5). A session of 15 passages was completed in ~30 min (incl. 1 min of rest between passages).

A reading time of 55 s per text may represent a rather long duration in case of demanding tasks (e.g., any manipulation of passages display or layout that increases cognitive load and/or visual stress). In such cases, reading performance may be affected by fatigue, making it challenging to test multiple experimental conditions. A WRRT based on shorter equivalent passages would solve this issue. Shorter passages could also be helpful in studies involving the elderly or neuropsychological patients, who may present with attentional deficits, get quickly tired, or present with cognitive fatigability (e.g., Möller et al., 2014).

Therefore, the aim of the present study was to develop a Short Italian Wilkins Rate of Reading Test (SI-WRRT), a ready-to-print Italian version using 5 lines instead of the 10-line passages (as in the standard Italian WRRT, or in the original WRRT in English). While maintaining the same 15 high-frequency words and number of passages (15) of the standard Italian WRRT, the present study examined whether the 5-line passages retained the same characteristics of the 10-line ones in terms of equivalence, practice and fatigue effects, and test-retest reliability. Although the 5-line passages originated from the standard Italian WRRT (Zeri et al., 2023), the equivalence was re-assessed, because the smaller layout size of the 5-line text (and resulting shorter reading time) may introduce reading speed differences across passages.

## 2 Materials and methods

All procedures and the use of optometric tests and reading materials were undertaken in accordance with the Declaration of Helsinki and approved by the Ethics Committee of the University of Milano-Bicocca (Prot. N. 0398635 del 30/10/2023 – UOR: 003406).

### 2.1 Participants

Higher-education students from the University of Milano-Bicocca (Milan, Italy) were recruited. A thorough eye examination and vision assessment, based on a standard optometric examination and a standard assessment of near functional vision during reading, were carried out to include only participants capable of fluently reading at near (cf. inclusion criteria outlined in Table 1), to ensure a reliable assessment of equivalence across the 15 SI-WRRT passages. All volunteers provided written informed consent. G^*^Power software (www.gpower.hhu.de) was used to determine the sample size for a repeated-measures design (ANOVA and paired comparisons) and test-retest reliability. An effect size of 0.66 was calculated for a reading speed difference between two measurements of 10 wpm, which is a clinically relevant difference in optometry and ophthalmology (Altpeter et al., 2015; Kaltenegger et al., 2019; Stöhr et al., 2024), consistent with previous measures obtained from our laboratory (unpublished data). A difference of <10 wpm indicates comparable reading speeds for different texts. Using a standard α = 0.05 and a power (1-β) = 0.80 returned a minimum sample size of *n* = 21. However, in the present study we adopted a “conservative approach” and decided to double the sample size (*n* = 42) to increase statistical power, that is, the probability of correctly rejecting the null hypothesis (i.e., equivalence of passages), hence increasing the probability of identifying the presence of non-equivalent passages, if there are any. Based on this, 42 participants were enrolled. One participant had to be excluded due to the presence of developmental dyslexia identified during the medical history assessment. Another one dropped out of the study. There were no participants with visual profiles unsuitable for reading at near. Hence, our final sample included 40 participants (23 females and 17 males; mean age: 24.2 ± 3.7 years, range 19.0 – 35.0; mean years of education: 16.0 ± 2.0, range 13 – 21 years) all of whom returned for retesting after 2 weeks.

**Table 1:** Inclusion criteria for participants enrolled in the study (see Section 2.3).

**Inclusion criteria**
Native Italian speakers
Absence of known reading disability
No ocular pathology
No significant ocular motility or binocular vision anomalies (including strabismus)
Monocular best-corrected visual acuity (BCVA) at distance ≤ 0.10 logMAR in each eye
Near point of convergence ≤ 10 cm
Stereoscopic acuity ≤ 80 arcsec
Binocular amplitude of accommodation ≥ 8 D
Binocular accommodative facility with ± 2.00D lenses ≥ 5 cpm
Reading acuity ≤ 0.2 logRAD at the Radner Reading Charts
Ability to read, comprehend, and sign the informed consent form

### 2.2 Development of the Short Italian WRRT (SI-WRRT)

The SI-WRRT builds upon the standard Italian WRRT (Zeri et al., 2023). The latter is made up of 15 10-line passages, each line of which contains 15 high-frequency words [i.e., belonging to the 2,000 most frequent words of the Italian language; cf. “fundamental words” in De Mauro (2016)], the same words in each line, arranged in random order. The words are: *di* [of], *ha* [has], *si* [third person reflexive pronoun, used in reflexive verbs], *la* [“the” for feminine nouns], *amo* [I love], *che* [that/which], *con* [with], *era* [was], *fai* [(you) do], *non* [not], *per* [for], *una* [“a” for feminine nouns], *anno* [year], *sono* [am/are], and *uomo* [man]. Each word appears only once per line and once in a specific serial position (i.e., from 1 to 15). Additionally, the last word in each line is different from the first word in the next line, and all lines across passages are different. The typesetting conforms to the typographic specifications of the original WRRT (Wilkins et al., 1996), featuring Times New Roman font, 9-point print size (i.e., 0.5 logMAR at a viewing distance of 40 cm, whereby logMAR is the logarithm of the minimum angle of resolution), single-spaced lines (3.15 mm), and 4-point horizontal spacing between words. For the creation of the 5-line passages, each 10-line passage from the standard Italian WRRT was split into two halves, resulting in a total of thirty 5-line passages, 15 of which (labelled with consecutive lowercase letters from “a” to “o”) were used in the present study. The final layout of each passage is a paragraph 72.5 mm wide and 17.0 mm high, containing 15 words per line x 5 lines. Each passage is arranged on a separate page of a Microsoft Word file (www.microsoft.com) and printed at 1,200 dpi resolution. The set of ready-to-print passages is available in the Supplementary material.

### 2.3 Vision assessment

Participants underwent a preliminary comprehensive eye examination and vision assessment at the Research Centre in Optics and Optometry of the University of Milano Bicocca (COMiB). Ocular pathologies, subjective refraction, best-corrected visual acuity (BCVA), ocular motility, accommodation amplitude and facility, near point of convergence, and stereoacuity were assessed using specific standard optometric tests (whose details are reported in the Supplementary material).

Maximum reading speed (MRS), critical print size (CPS), and reading acuity (RA)—i.e., parameters that quantify near functional vision during reading (Calabrèse et al., 2016; Radner, 2016)—were measured binocularly using the standardised Italian version of the Radner Reading Charts (Radner et al., 1998; Calossi et al., 2014) at 40 cm. This test is a “sentence optotypes” chart consisting of 15 different 3-line meaningful sentences printed on cards with progressively smaller print sizes. The print size decreases logarithmically by 0.1 logMAR from the first to the 15th sentence, ranging from 1.2 to −0.2 logMAR. The number, length in characters, and frequency of use of the words are comparable across sentences, as well as syntactical construction (Radner et al., 1998). MRS is the fastest speed achieved across large print sizes representing the plateau of the reading speed curve plotted against print size, before the speed declines beyond the CPS. The CPS is the smallest print size at which one can still read at their maximum speed. RA corresponds to the smallest print size at which one can read a whole sentence. It is measured as logRAD, i.e., the logarithm of the Reading Acuity Determination (RAD), which is equivalent to the print size measured in logMAR adjusted for the reading errors made in the last sentence read entirely. Based on the outcomes of the standard optometric tests (see Section 3.1 in the Results), 29 participants kept their habitual spectacles or contact lenses, while 11 did not need any refraction correction to read the Radner Reading Charts.

### 2.4 SI-WRRT administration

A test and a retest session took place 2 weeks apart in the same room. Participants were assessed individually. Tests and retests were carried out using the same procedure, following detailed written instructions that were read to participants (see the file “De Luca et al. Front. Psychol. 2024 Short Italian WRRT text passages.pdf” in the Supplementary material). The same examiner, who was different from the one who carried out the vision assessment and blind to its outcome, carried out the test and retest sessions for each participant. Each passage was displayed on a single page on a reading desk at a viewing distance of 40 cm. Participants read the passages under photopic conditions (550 ± 50 lux, measured by a luxmeter HT307, HT Italia; Faenza, Italy) with an average luminance of the paper surface (eight measurements) of 135 ± 11 cd/m^2^ (Chroma metre CS 100 A; Minolta; Osaka, Japan). The refraction correction for participants was the same as in the Radner Reading Charts (see section above).

Participants were asked to read the entire passage aloud as fluently and accurately as possible, with an interval of 1 min between passages. The presentation order of the passages from “a” to “o” was randomised across participants, and the reading was recorded digitally. Reading speed for correctly read words (wpm) and accuracy (percentage of reading errors) were measured offline, using Audacity (www.audacityteam.org) to replay the recordings and detect the reading onset and offset by examining the acoustic spectrum. Reading errors were scored according to the same criteria used for the 10-line passages: word substitution (replacing a word with another), word omission (skipping a word), line omission, word insertion (repeating the previously uttered word or inserting another word), and production of a non-word (a pronounceable string of letters that is not in the lexicon), with each error scored as “1.”

### 2.5 Data analysis

The jamovi package (www.jamovi.org) was used to compute the descriptive statistics for the results of the vision assessment and the SI-WRRT (reading speed as wpm, and reading accuracy as percentage of reading errors), as well as all other analyses. The normal distribution of reading speed and accuracy data was checked using the Shapiro-Wilk test. Repeated-measures analyses were run using an ANOVA or a Friedman test, depending on the normality of the data distribution, to assess the equivalence across the 15 passages (from “a” to “o”), and any practice and fatigue effects of consecutive readings (i.e., reading order). *T*-tests were run as (two-tailed) *post-hoc* tests in case of parametric analyses. Durbin-Conover tests were run as *post-hoc* tests in case of non-parametric analyses. *Post-hoc* tests for practice and fatigue effects were run within two separate time-windows, i.e., the first seven readings and the last seven readings, respectively, based on where such effects were expected (e.g., cf. Zeri et al., 2023). For reading speed, the test-retest reliability was computed using the Intraclass Correlation Coefficient (ICC) based on a “two-way mixed effects, consistency type, single measure” model (Koo and Li, 2016), and 95% confidence intervals were calculated. Paired *t*-tests and Wilcoxon tests (for speed and accuracy data, respectively) were carried out for test-retest comparisons. Bonferroni correction for multiple testing (as calculated by jamovi) was applied in all analyses, and corrected *p*-values were reported.

## 3 Results

### 3.1 Vision assessment

All participants showed adequate eye and visual function including visual acuity, accommodation, and binocular vision, as well as good near functional vision during reading. Twenty-nine participants showed negligible differences with respect to the subjective refraction measured during the optometric assessment, therefore, during both the Radner Reading Charts administration and the SI-WRRT reading session, they kept their habitual refractive correction (spectacles or contact lenses), as normally used for reading and studying. Among the remaining 11 participants, who did not wear any refractive correction, nine were emmetropes, and two had negligible myopic refractive errors that did not necessitate correction. Group results and additional details are provided in Supplementary Table 1.

Regarding the Radner Reading Charts (see Table 2), both the participants' RA (-0.1 logRAD, on average) and CPS (0.1 logMAR, on average) corresponded to a print size smaller than that of the WRRT (0.5 logMAR). Taken together, these results ensure that all participants could successfully read the SI-WRRT at their maximum speed without any limitations due to print size.

**Table 2:** Radner Reading Charts used for assessing near functional vision during reading.

**Radner Reading Charts parameter**	**Mean**	**Median**	**SD**	**Min**	**Max**
RA (logRAD)	−0.1	−0.1	0.1	−0.2	0.1
CPS (logMAR)	0.1	0.1	0.1	−0.1	0.3
MRS (wpm)	208.4	210.1	25.7	164.3	255.9

Results indicate that participants were capable of fluently reading at near, which ensured a reliable assessment of equivalence across the SI-WRRT 15 passages.

RA, reading acuity; logRAD, logarithm of the Reading Acuity Determination (RAD), i.e., reading acuity equivalent of logMAR; CPS, critical print size; MRS, maximum reading speed; wpm, words per minute; SD, standard deviation.

### 3.2 SI-WRRT

#### 3.2.1 Equivalence across passages

**Figure 1:**
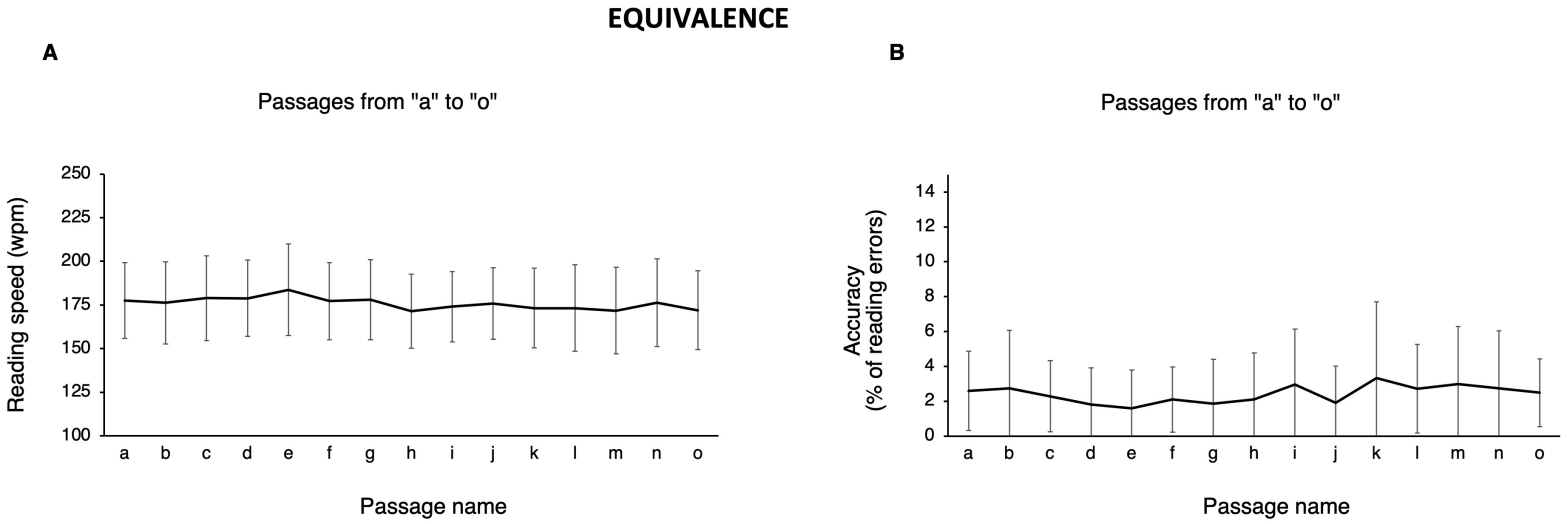
Equivalence across passages. Mean (error bars: standard deviation) of **(A)** reading speed (words per minute; wpm) and **(B)** accuracy (% of reading errors) for the 15 passages (“a”—“o”).

Figure 1 and Table 3 present the descriptive statistics of reading speed and accuracy for the 15 passages (test session).

**Table 3:** Equivalence across passages.

	**Reading speed (wpm)**				**Reading accuracy (% of reading errors)**				
**Passage name**	**Mean**	**SD**	**Min**	**Max**	**Mean**	**Median**	**SD**	**Min**	**Max**
a	177.5	21.7	135.1	220.4	2.6	2.4	2.3	0.0	10.9
b	176.2	23.7	134.6	223.1	2.7	1.7	3.3	0.0	15.2
c	179.0	24.2	129.2	231.9	2.3	1.7	2.0	0.0	10.3
d	178.9	21.8	136.1	232.6	1.8	1.4	2.1	0.0	10.9
e	183.8	26.3	138.3	239.9	1.6	1.0	2.2	0.0	9.7
f	177.3	22.1	129.9	236.5	2.1	2.0	1.9	0.0	6.9
g	178.0	23.0	129.0	242.1	1.9	1.3	2.5	0.0	8.6
h	171.4	21.2	131.2	226.1	2.1	1.4	2.7	0.0	14.6
i	174.0	20.1	137.7	223.1	2.9	2.1	3.2	0.0	10.9
j	175.8	20.6	134.5	224.6	1.9	1.4	2.1	0.0	8.0
k	173.2	22.8	125.2	233.3	3.3	1.7	4.4	0.0	25.0
l	173.3	24.7	117.0	229.9	2.7	2.0	2.5	0.0	9.4
m	171.8	24.8	120.0	228.0	3.0	2.1	3.3	0.0	16.2
n	176.4	25.2	126.4	251.8	2.7	2.0	3.3	0.0	16.2
o	172.0	22.5	128.1	225.4	2.5	2.7	1.9	0.0	7.0

Mean, standard deviation (SD), and range (Min – Max) for reading speed (words per minute; wpm) and accuracy (% of reading errors) for the 15 passages (“a”—“o”).

Reading speed showed a normal distribution for all passages (Shapiro-Wilk test: *p* > 0.05) except for passage “n” (Shapiro-Wilk test: *p* = 0.005). Reading speed across passages was 175.9 ± 3.4 wpm (range 171.4 – 183.8). Repeated-measures ANOVA revealed a statistically significant difference in reading speed across passages [*F*_(1, 14)_ = 3.74; *p* < 0.001]. *Post-hoc* testing identified five significant comparisons (all *p*-values <0.05 after applying Bonferroni correction). All significant comparisons involved passage “e” (paired with “h,” “i,” “k,” “m,” and “o”). In fact, passage “e” was on average 11.3 wpm (range 9.8 – 12.4 wpm) faster than these passages, a difference that is also clinically relevant (cf. Section 2.1). The average of non-significant differences with passage “e” was 6.9 wpm (range 4.8 – 10.5 wpm, see below). Despite its faster reading speed, passage “e” did not compromise accuracy, as the error rate was only 1.6% (i.e., lower than other passages). Passage “e” also showed a clinically relevant difference of 10.5 wpm with passage “l,” but the comparison was not significant. Excluding passage “e” returned an average reading speed across passages of 175.3 ± 2.7 wpm (range 171.4 – 179.0).

For reading accuracy, no passage showed a normal distribution (Shapiro-Wilk test: all *p*-values <0.05). The average percentage of reading errors was 2.4% ± 0.5 (median 2.5%; range 1.6 – 3.3%). The Friedman test indicated a significant difference across passages (*r* = 27.4; *p* = 0.017). *Post-hoc* testing did not show any significant paired comparison. Excluding passage “e,” which showed non-equivalence in reading speed, resulted in an error rate of 2.5% ± 0.5, that is, an overall accuracy of 97.5%. Finally, reading speed was not associated with reading accuracy (Spearman's Rho = −0.14, *p* = 0.387).

#### 3.2.2 Practice and fatigue effects

**Figure 2:**
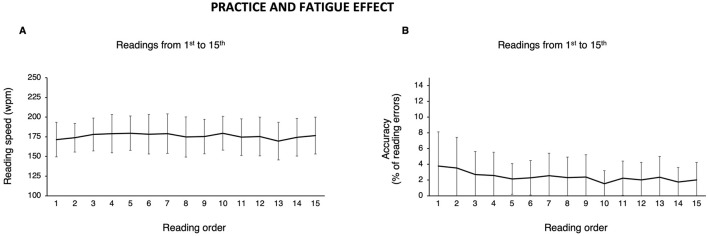
Practice and fatigue effects. Mean (error bars: standard deviation) of **(A)** reading speed (words per minute; wpm) and **(B)** accuracy (% of reading errors) as a function of the reading order.

Figure 2 and Table 4 show reading speed and accuracy as a function of reading order (test session).

**Table 4:** Practice and fatigue effects.

	**Reading speed (wpm)**				**Reading accuracy (% of reading errors)**				
**Reading order**	**Mean**	**SD**	**Min**	**Max**	**Mean**	**Median**	**SD**	**Min**	**Max**
1st reading	171.4	21.9	138.3	231.0	3.8	2.7	4.3	0.0	25.0
2nd reading	173.7	18.2	139.2	218.9	3.5	2.1	3.9	0.0	16.2
3rd reading	177.9	20.7	129.2	228.0	2.7	2.1	2.9	0.0	16.2
4th reading	178.9	24.2	138.1	232.6	2.6	1.4	3.0	0.0	13.4
5th reading	179.6	21.9	146.0	239.9	2.1	1.7	2.0	0.0	8.0
6th reading	178.3	25.0	134.5	236.5	2.3	2.0	2.2	0.0	7.0
7th reading	179.1	25.2	137.7	242.1	2.5	2.0	2.9	0.0	10.9
8th reading	174.8	25.5	134.0	231.9	2.3	1.4	2.6	0.0	11.8
9th reading	175.3	21.9	129.0	227.5	2.4	1.4	2.8	0.0	10.9
10th reading	179.5	21.5	129.9	235.5	1.5	1.3	1.6	0.0	4.9
11th reading	174.5	23.3	128.1	223.1	2.2	1.7	2.2	0.0	9.3
12th reading	175.4	24.6	126.4	251.8	2.0	1.4	2.2	0.0	8.6
13th reading	169.5	23.9	120.0	222.0	2.4	2.1	2.6	0.0	14.6
14th reading	174.4	24.0	117.0	229.9	1.8	1.3	1.9	0.0	7.0
15th reading	176.6	23.4	125.2	233.3	2.0	1.3	2.2	0.0	7.1

Mean, standard deviation (SD), and range (Min – Max) for reading speed (words per minute; wpm) and accuracy (% of reading errors) for the readings, from the 1st to the 15th.

Reading speed showed a normal distribution for all readings (Shapiro-Wilk test: *p* > 0.05) except for the 1st, 4th, 5th, and 7th readings (Shapiro-Wilk test: *p* < 0.05). Reading speed across all readings was 175.9 ± 3.02 wpm (range 169.5 – 179.6). Repeated-measures ANOVA revealed a significant difference across readings [*F*_(1, 14)_ = 2.94; *p* < 0.001], indicating an effect of reading order. As regards the practice effect, *post-hoc* testing in the first time-window (i.e., across the first seven readings) identified a single significant comparison between the 1st and the 5th reading (whereby the 1st reading was slower than the 5th; *p* = 0.047 after applying Bonferroni correction), although the difference (8.2 wpm) was not clinically relevant. As regards the fatigue effect, *post-hoc* testing in the second time-window (i.e., across the last seven readings) identified two significant comparisons: between the 13th and the 10th reading, and between the 13th and the 15th reading, whereby the 13th was slower than both (see Table 4; *p* < 0.001 and *p* = 0.021, respectively after applying Bonferroni correction). However, only the difference between the 13th and the 10th reading was also clinically relevant. Reading speed between the 3rd and the 10th reading (i.e., the plateau visible in Figure 2A, before performance gets slower) was 177.9 ± 1.9 wpm (range 174.8 – 179.6).

As regards reading accuracy, none of the readings showed a normal distribution (Shapiro-Wilk test: all *p*-values <0.01). The percentage of reading errors was 2.4% ± 0.6 (median 2.3%; range 1.5 – 3.8%). Friedman test revealed a significant difference across readings (*r* = 30.9; *p* = 0.006), but *post-hoc* testing showed no significant comparisons in either time-windows.

#### 3.2.3 Test-retest reliability

**Figure 3:**
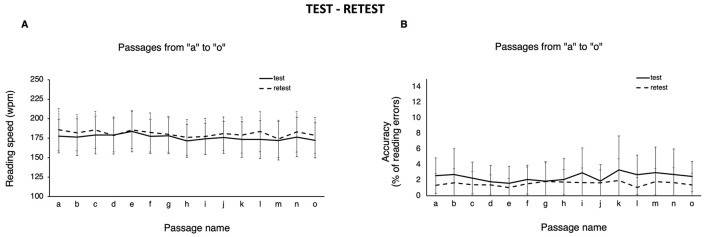
Test-retest. Mean (error bars: standard deviation) of **(A)** reading speed (words per minute; wpm) and **(B)** accuracy (% of reading errors) for the 15 passages (“a”—“o”), separately for test (continuous line) and retest (dashed line) sessions.

All participants underwent a retest 2 weeks after the initial session. Figure 3 shows the results of both test and retest sessions, presenting data about passages from “a” to “o,” separately for reading speed and accuracy. Descriptive statistics and paired comparisons between test and retest sessions for reading speed and accuracy, along with Intraclass Correlation Coefficients (ICC) specifically for reading speed, are provided in Table 5. Generally, retest performance showed an improvement in both reading speed (180.7 ± 3.6 wpm) and accuracy (1.6% ± 0.3). Reading speed was faster in 14 out of the 15 passages, with a significant increase observed only in one passage (“l;” *p* = 0.014 after applying Bonferroni correction), which also represented a clinically relevant difference (10.3 wpm). The other differences were neither statistically significant, nor clinically relevant. Accuracy improved for all passages, but only three (“a,” “l,” and “o”) showed a significant difference (1.2%, *p* = 0.008; 1.6%, *p* = 0.004; 1.1%, *p* = 0.041, respectively). The ICC for reading speed (wpm), the standard metric for WRRT, indicated moderate-to-good reliability for all passages (range 0.67 – 0.82).

**Table 5:** Test-retest reliability separately for reading speed (words per minute; wpm) and accuracy (% of reading errors).

	**Reading speed (wpm)**				**Reading accuracy (% of reading errors)**		
**Passage name**	**Test Mean** ±**SD**	**Retest Mean** ±**SD**	**Paired** ***t*****-test comparison**	**ICC (95% CI)**	**Test Mean** ±**SD**	**Retest Mean** ±**SD**	**Wilcoxon test comparison**
a	177.5 ± 21.7	185.8 ± 27.1	n.s.	0.74^*^ (0.56 – 0.85)	2.6 ± 2.3	1.4 ± 1.5	***p*** **=** **0.008**
b	176.2 ± 23.7	181.8 ± 23.2	n.s.	0.78^*^ (0.62 – 0.88)	2.7 ± 3.3	1.7 ± 1.8	n.s.
c	179.0 ± 24.2	185.5 ± 23.7	n.s.	0.73^*^ (0.55 – 0.85)	2.3 ± 2.0	1.4 ± 1.7	n.s.
d	178.9 ± 21.8	178.3 ± 24.0	n.s.	0.76^*^ (0.60 – 0.87)	1.8 ± 2.1	1.4 ± 1.3	n.s.
e	183.8 ± 26.3	185.5 ± 23.9	n.s.	0.68^*^ (0.47 – 0.82)	1.6 ± 2.2	1.1 ± 1.2	n.s.
f	177.3 ± 22.1	182.3 ± 25.2	n.s.	0.74^*^ (0.56 – 0.86)	2.1 ± 1.9	1.6 ± 2.1	n.s.
g	178.0 ± 23.0	179.9 ± 23.0	n.s.	0.82^*^ (0.68 – 0.90)	1.9 ± 2.5	1.8 ± 2.4	n.s.
h	171.4 ± 21.2	176.0 ± 23.0	n.s.	0.81^*^ (0.67 – 0.89)	2.1 ± 2.7	1.8 ± 1.6	n.s.
i	174.0 ± 20.1	177.1 ± 23.3	n.s.	0.81^*^ (0.67 – 0.90)	2.9 ± 3.2	1.7 ± 1.9	n.s.
j	175.8 ± 20.6	180.7 ± 21.6	n.s.	0.69^*^ (0.48 – 0.82)	1.9 ± 2.1	1.7 ± 1.6	n.s.
k	173.2 ± 22.8	178.8 ± 23.1	n.s.	0.73^*^ (0.54 – 0.85)	3.3 ± 4.4	2.0 ± 2.8	n.s.
l	173.3 ± 24.7	183.6 ± 25.8	***p*** **=** **0.014**	0.74^*^ (0.56 – 0.86)	2.7 ± 2.5	1.1 ± 1.3	***p*** **=** **0.004**
m	171.8 ± 24.8	173.9 ± 23.8	n.s.	0.72^*^ (0.53 – 0.84)	3.0 ± 3.3	1.8 ± 1.7	n.s.
n	176.4 ± 25.2	183.0 ± 25.9	n.s.	0.67^*^ (0.45 – 0.81)	2.7 ± 3.3	1.7 ± 1.9	n.s.
o	172.0 ± 22.5	178.5 ± 23.0	n.s.	0.77^*^ (0.61 – 0.87)	2.5 ± 1.9	1.4 ± 1.5	***p*** **=** **0.041**

The table reports descriptive statistics (mean and standard deviations) and p-values of paired comparisons between test and retest. Intraclass Correlation Coefficient (ICC) and confidence intervals (CIs) between test and retest (calculated with two-way mixed effects model, consistency, and single measure) are presented for reading speed—the standard metric for WRRT. Significant p-values correspond to corrected p-values after applying Bonferroni correction.

^*^ICC significant with p <0.001.

## 4 Discussion

Reading performance on parallel forms of texts is commonly used by clinicians and researchers in vision science as a tool to reliably assess (by measuring reading speed) the effectiveness of interventions in patients with vision deficits, developmental reading disabilities, or visual perception impairments following acquired brain lesion (e.g., Bailey and Lakshminaryanan, 1997; Spitzyna et al., 2007; Tilanus et al., 2019). Recently, it has been proposed that the WRRT may be considered as a RAN (rapid automatized naming) test (Gilchrist et al., 2021), i.e., a task commonly used in neuropsychology of developmental reading disorders. Both tests share rapid processing from left to right of arrays of recurrent familiar stimuli, although WRRT is based on reading recurrent unrelated words, while RAN is based on naming recurrent items (digits, coloured squares, or other visual stimuli arranged in arrays; Denckla and Rudel, 1974). The proposal has a heuristic value, since studies in the field of developmental neuropsychology reported that RAN tasks are associated with reading fluency (e.g., Georgiou et al., 2013; Landerl et al., 2019) and reading deficits (e.g., Denckla and Rudel, 1976; Norton and Wolf, 2012). In ophthalmology and optometry, reading performance allows the evaluation of reading acuity, critical print size, reading speed, and maximum reading speed. These parameters are used to measure the outcomes of interventions such as cataract surgery with lens implantation, presbyopia correction, determination of magnification need under different visual conditions and low vision aids, prismatic corrections, eye exercises, or refractive modifications (Alió et al., 2011; Buckhurst et al., 2012; Crossland et al., 2019; O'Leary and Evans, 2006; Zeri et al., 2018). These parameters are also commonly adopted to evaluate interventions for the improvement of reading performance in patients with developmental dyslexia (Tilanus et al., 2019; Wilkins et al., 1996) and hemianopia (Daibert-Nido et al., 2021; see also Schuett et al., 2008 for a review on hemianopic dyslexia).

### 4.1 Parallel forms of texts in vision science

The most common tests measuring these parameters are serial texts such as the Bailey-Lovie Reading Sentence Chart (Bailey and Lovie, 1980), MNRead Acuity Charts (Mansfield et al., 1993; Ahn et al., 1995), and Radner Reading Charts (Radner et al., 1998). These tests are based on very short texts (1–3 lines) made up of either unrelated words (as in the Bailey-Lovie chart) or continuous text (i.e., sentences with a meaning, as in the MNRead and Radner charts), printed with progressively smaller print size. Other common materials suitable for repeated measurements of reading speed are represented by longer texts, such as the passages of the New International Reading Speed Texts (IreST; Hahn et al., 2006; Trauzettel-Klosinski et al., 2012), which are printed with a fixed print size, and another version of the Radner charts made up of long paragraphs (i.e., texts longer than the sentences of the original charts; Radner et al., 2016). Standardised versions of Radner, MNRead, and IreST exist in different languages (see Rubin, 2013 and Radner, 2017, for reviews). Additionally, the IreST is matched for psycholinguistic variables and syntactic complexity across languages.

All tests have advantages and disadvantages. Short texts with a progressive reduction in print size are commonly used to determine the outcome of treatments and interventions, as mentioned above. They accurately assess CPS and RA very quickly, but may be less accurate in measuring speed, unless performance is digitally recorded, or examiners are thoroughly trained (see Radner et al., 2017 for a discussion on the accuracy of reading time measurements). It has been observed that short texts (e.g., texts no longer than 60 characters; cf. Rubin, 2013) may inaccurately measure reading speed due to several factors, including an examiner's reaction time in starting and stopping the stopwatch, as well as pauses and self-corrections by the reader. Long texts are commonly adopted to measure sustained reading and functional reading speed (typically assessed in low vision patients). It has been claimed that long texts yield more reliable reading speed measures and should therefore be preferred in repeated measurements (e.g., Kortuem et al., 2021). However, even after thorough linguistic matching of text complexity and equivalence in the number of characters and syllables, number and length of words, as well as words position and overall layout, long texts may still not yield comparable reading measures. In fact, differences may remain undetected unless the texts are statistically validated (e.g., Brussee et al., 2015). For example, Radner et al. (2016) found unexpected results (i.e., non-comparable reading speeds) in the development of texts for their long paragraphs that were built to have equivalent readability scores. It is possible that other cognitive factors (incl. emotional and attentional factors; cf. Radner et al., 2016) played a role simply because of the presence of syntax, semantics, and text meaning. In other words, even if most texts are very simple and suitable for 6th-grade readers (e.g., Trauzettel-Klosinski et al., 2012), the minimal literacy demand may not be sufficient to avoid uncontrolled effects, because the presence of a semantic context is potentially capable of influencing reading speed (cf. Rubin, 2013, about the semantic context controversy). In addition, it has been shown that short equivalent MNRead sentences generated by algorithms under strict linguistic and layout constraints may determine non-comparable reading performances, thus prompting a recommendation to screen new sentences before using them (Mansfield et al., 2019).

Therefore, matching linguistic variables makes it challenging to generate and validate a series of parallel meaningful passages. An interesting solution adopted in vision science is to neutralise/minimise the role of syntax and semantics at the sentence-level by using the same set of shuffled unrelated words across lines and passages. This approach was introduced in the original WRRT by Wilkins et al. (1996), where 15 high frequency words are randomised across 10-line 15-word passages. In this vein, the standard Italian WRRT (Zeri et al., 2023) was generated by transliterating (not directly translating) the original WRRT and expanded the number of passages, demonstrating the equivalence of 15 10-line passages of unrelated words. Therefore, the standard Italian WRRT provided suitable material for studies with repeated-measures designs involving multiple conditions or measurements over time (e.g., baseline and follow-ups). The reading speed observed in Italian participants who read the standard Italian WRRT (167.3 ± 1.6 wpm) was consistent with the results obtained in studies measuring reading speed (see Brysbaert, 2019, for a meta-analysis based on data from several languages; see also Gilchrist et al., 2021). The standard Italian WRRT showed a practice effect, which expired after the first reading, while there was no fatigue effect. However, studies involving the elderly or brain-damaged patients may be vulnerable to fatigue when using long texts to test multiple conditions. For instance, each passage of the standard Italian WRRT (unrelated words) takes about 55 s to be read, while each passage of the Italian version of the IReST (meaningful sentences) takes about 45 s. Therefore, the need for a shorter WRRT for studies involving patients who may get tired more rapidly and/or have attentional deficits has prompted the development of a shorter test, the SI-WRRT (the focus of the present study), based on the same principles and constraints adopted in the original WRRT (as well as in the standard Italian WRRT).

### 4.2 Equivalence across SI-WRRT passages

The present study showed that the passages of the SI-WRRT are equivalent to each other, except one. Specifically, passage “e” showed a faster reading speed compared to the others (see Table 3). Such discrepancy was both statistically significant and clinically relevant with respect to five passages, and only clinically relevant with respect to another one. However, the average reading speed across passages remains comparable, whether including passage “e” (175.9 wpm) or excluding it (175.3 wpm) from the testing set. Furthermore, there is no speed/accuracy trade-off, as passage “e” was not read faster at the expense of accuracy. Overall, there is no association between reading speed and accuracy in the whole SI-WRRT. Indeed, accuracy was higher (but not statistically different) for passage “e” (1.6% error rate). Therefore, as passage “e” is the only non-equivalent one, we strongly recommend excluding it from the items used for repeated measures, and instead using it as a familiarisation item (see practice effect in Section 4.3). This finding challenges the notion that simply rearranging the positions of unrelated words automatically generates equivalent passages (Wilkins et al., 1996; Gilchrist et al., 2021). It also underscores the importance of testing the equivalence of reading speed and accuracy for newly generated passages of unrelated words, even if they are excerpts derived from previously tested and validated passages (as it is the case with the SI–WRRT, which was derived from the standard Italian WRRT), before using them in research or in clinical practice.

As mentioned above, the average reading speed observed for a passage of the SI-WRRT is 175.3 wpm. This value corresponds to a reading time of 25.5 s, which is about half the time needed for the 10-line version (54.9 s). A session of 14 passages is completed in ~19 min (including 1 min of rest between passages). The difference in reading speed between the 5-line and the 10-line test is 8.6 wpm, a value below the clinically relevant difference (i.e., 10 wpm). Indeed, both the 5-line and 10-line speed values lie within the range indicated in the above-mentioned review by Brysbaert (2019). However, the slightly faster speed for the 5-line passages may be explained by the reduction in visual crowding in the test. The original idea of Wilkins et al. (1996) was to create a test that maximised visual stress (with crowded word arrangement on a line, and a tight line spacing), along with neutralising/minimising syntactical and semantical implications. Reducing the number of lines in the test from 10 to 5 could have diminished visual stress by reducing the layout size of the text, and thus the density of the page. This hypothesis could be tested in future studies by adding flankers (i.e., lines of text that enhance the density of the layout, but are not read by participants).

Differently from the vision assessment in the 10-line study, in the present study we also administered the Italian version of the Radner Reading Charts (Calossi et al., 2014) to quantify near functional vision during reading. The participants' RA and CPS were better than the visual capacity required by the WRRT print-size, which is supra-threshold (0.5 logMAR) with respect to the reading acuity (-0.1 logRAD) and CPS (0.1 logMAR) assessed in our sample. Therefore, using this test allowed us to ascertain that participants were in optimal visual reading conditions and could read the WRRT at their maximum speed without any limitations due to the print size of passages.

The accuracy is almost identical in the two versions (2.5% ± 0.3 and 2.4% ± 0.5 error rates, for standard WRRT and SI-WRRT, respectively, corresponding to <4 and <2 words, respectively). Reading accuracy measurements should always be part of a protocol assessing reading speed. While reading errors are accounted for by default when reading optotypes (as the criterion to go from a given print size to a smaller one relies on correctly reading a sentence), long readings need to score reading errors during online performance. However, this has not always been accomplished in the past (see Brussee et al., 2014, for a review). In the present study, reading speed was determined by analysing digital audio recordings, which enabled an accurate measurement of both reading errors and reading time. This allowed a reliable computation of reading speed based on correctly read words, as per WRRT principles (Wilkins et al., 1996). In the present study, error rate was very low (range 1.6 – 3.3%). Since error rate may not be negligible in patients with low vision, neuropsychological deficits due to acquired brain lesions, or developmental reading disorders, measuring reading errors should always be part of the procedure to reliably measure reading speed.

### 4.3 Practice and fatigue effects

Contrary to the findings reported in the standard Italian WRRT (Zeri et al., 2023), this study revealed a less pronounced practice effect for reading speed. The only significant difference occurred between the 1st and the 5th reading, which was not clinically relevant. Overall, results indicate a slow reading speed improvement characterised by progressively faster speeds across the initial five readings, with the first two readings slower than the following ones, and a plateau from the 3rd reading onwards (see Figure 2A and Table 4). One may presume that in vulnerable populations, such as the elderly or neuropsychological patients, statistically significant differences may emerge more readily (see section 4.5). Hence, we confirm the need to familiarise with the test (i.e., reading at least one passage) before proceeding with consecutive readings for experimental or clinical purposes (i.e., repeated measures). Therefore, passage “e” can be used to this purpose (see Section 4.2). This prevents from biassing the interpretation of the effect of interventions (e.g., Allen et al., 2012). As regards accuracy, there were neither significant effects, nor clinically relevant differences.

As for the fatigue effect, significant differences occurred between the 10th and the 13th reading, and between the 13th and the 15th (only the former being clinically relevant). Figure 2A shows a progressive decline in reading speed (mostly non-significant) evident after the 10th reading (179.5 wpm) down to the dip by the 13th reading (169.5 wpm). The last two readings of Figure 2A can be interpreted as a kind of “relief-effect” [an identical trend, although not significant, was found in Zeri et al. (2023)]. Therefore, both the 10- and the 5-line versions are potentially suitable to assess prolonged reading for at least 10 consecutive readings in the same session. Once more, studies on the elderly or patients with specific issues are needed to determine fatigue effects in populations different from the one tested in the present study (i.e., healthy controls; see Section 4.5).

### 4.4 Test-retest reliability

In line with previous research on the WRRT (e.g., Stifter et al., 2004), the test-retest reliability was assessed. In both the present study and in previous ones (e.g., Wilkins et al., 1996; Zeri et al., 2023), results showed a generally improved performance in both reading speed and accuracy at retest. This can be easily explained by a slight learning process. Importantly, as regards reading speed, which is the standard metric for WRRT, 14 passages were neither significantly faster, nor showed a clinically relevant difference (i.e., >10 wpm) between test and retest. One passage (“l”) was significantly faster at retest, and the difference (10.3 wpm) was clinically relevant. As regards accuracy, only three passages (“a,” “l,” and “o”) improved significantly, but the differences were negligible (range 1.1–1.6% of errors). Test-retest reliability showed moderate-to-good ICC for all passages, with the exception of passages “e,” “j,” and “n” (ICC = 0.68, 0.69, and 0.67, respectively). However, we would like to point out that, although correlations are usually computed in studies assessing the reliability of parallel forms across time, we suspect that such analysis may not be fully appropriate to assess the validity of texts for repeated measures, as in the present context (see Radner et al., 2016, for a similar position). This is because the availability of many equivalent texts makes it highly unlikely that an examiner would need to resort to the very same item twice at any time point. In other words, a passage would not be presented twice, since other equivalent passages are available. Therefore, as consistency across time depends on equivalence, assessing test-retest reliability would probably have little practical relevance.

### 4.5 Limitations and future directions

A possible limitation to the present study is that the trend observed in the practice and fatigue effect cannot be generalised to populations different from the sample tested here. Indeed, our study required the selection of participants with optimal reading capacity to determine the equivalence of the passages, and hence it was conducted on young, healthy individuals. However, the absence of a pronounced fatigue effect in our sample of readers does not prevent the potential occurrence of fatigue phenomena in more vulnerable populations (such as the elderly or neuropsychological patients, as well as readers with low vision, impaired reading ability, or attentional deficits). Therefore, it is essential that future studies will test the present materials in such populations.

Furthermore, future studies may test the hypothesis that the layout of the SI-WRRT leads to a reduction in reading time compared to the standard Italian WRRT because it decreases the density of the page, thereby reducing crowding. The reading materials to test this hypothesis may be based on the addition of flankers above and below the main text (i.e., lines of text that enhance the density of the layout, but are not read by participants).

## 5 Conclusions

The present study confirms the equivalence of 14 passages in the SI-WRRT, highlighting its usefulness for assessing reading speed in the elderly or neuropsychological patients in repeated-measures designs, due to the halved reading times of the 5-line passages with respect to the 10-line ones. As already suggested in Zeri et al. (2023), we reiterate the recommendation of providing a familiarisation item (i.e., giving participants a first item, which is not part of the assessment) before proceeding with the actual test for experimental or clinical purposes. Importantly, the non-equivalence of one passage underscores the need of a formal statistical validation before adopting random rearrangements of words to generate new passages.

## Data Availability

The datasets presented in this article are not readily available because of restrictions specified in the study consent-form, and conditions for approval from the local Ethics Committee, concerning participant confidentiality and privacy. Requests to access the datasets should be directed to the corresponding author, giuliacarlotta.rizzo@unimib.it.
