# Supplementary material for: Short Italian Wilkins Rate of Reading Test for repeated-measures designs in optometry and neuropsychology
Source: Front Psychol. 2024 Nov 19;15:1448817. doi: 10.3389/fpsyg.2024.1448817 (PMC11611593; doi:10.3389/fpsyg.2024.1448817)
Supplement: Supplementary file 1 [file Data_Sheet_1.pdf]

Supplementary material

Supplementary material (15 equivalent passages) to the paper

"Short Italian Wilkins Rate of Reading Test for repeated-measure designs in optometry and neuropsychology"

Maria De Luca[1†], Davide Nardo[2†], Giulia Carlotta Rizzo[3,4*], Roberta Daini[4,5], Silvia Tavazzi[3,4], and Fabrizio Zeri[3,4]

[1] IRCCS Fondazione Santa Lucia, Rome, Italy. [2] Department of Education, University of Roma Tre, Rome, Italy. [3] Department of Materials Science, University of Milano-Bicocca, Milan, Italy. [4] COMiB Research Centre in Optics and Optometry, University of Milano-Bicocca, Milan, Italy. [5] Department of Psychology, University of Milano-Bicocca, Milan, Italy.
[†] These authors have contributed equally to this work and share first authorship.
[*] Corresponding author

Istruzioni per il partecipante: "*Leggi ad alta voce nel modo più veloce e accurato possibile; in caso di errore, cerca di non correggerti e prosegui la lettura della matrice fino alla fine*".
[English translation: **Instructions for the participant:** "*Read aloud as quickly and accurately as possible; in case of an error, try not to correct it and continue reading the passage until the end*"].

uomo ha sono con una fai amo si di non per che la anno era
con fai per anno sono uomo si ha una la era di amo non che
per una non sono che con era la fai anno uomo si ha di amo
anno che si amo era per la di sono una ha fai non con uomo
la era uomo una di ha che non amo per sono con anno si fai

amo si che di non era uomo la con anno ha fai sono una per
di non per uomo una amo che anno ha sono era si la fai con
era anno ha si sono per con amo una la di non uomo che fai
si sono con una anno uomo di ha non che per la fai amo era
sono che amo per si la era una anno di fai uomo ha con non

anno fai non era che ha sono di si amo con una per la uomo
per era di con fai anno si che uomo ha la sono una non amo
con uomo si ha amo una la era per fai non che anno sono di
una amo la che per fai anno uomo di era si con non ha sono
la ha anno fai di non amo con sono uomo una era che per si

si anno uomo la che una ha per con di non sono fai amo era
anno che era ha amo sono con uomo si una la per non fai di
amo la con di si fai per era ha che uomo una anno sono non
con fai per anno era uomo che la sono amo di ha si non una
era di si fai uomo con amo una anno ha sono non la per che

ha amo di che per la anno sono non fai si era uomo una con
la si sono con fai di una non che era amo anno per ha uomo
una sono che amo non era si con per uomo fai di ha la anno
di con una non sono per uomo anno la si ha amo che era fai
che era non per la ha di fai una sono con uomo amo anno si

fai amo non anno uomo di una era sono ha con si che per la
era si di per fai sono che anno con uomo una la amo ha non
per uomo fai una con amo non la si che ha sono anno era di
la anno sono che si era ha fai di amo non per con uomo una
amo non era con ha per uomo sono una la anno di fai che si

di una la si anno che con ha non fai uomo amo era sono per
uomo con per fai di anno amo si che era sono una la non ha
sono era ha non una uomo la che per anno di con si amo fai
che di anno la amo si fai non era sono per uomo ha una con
non per con uomo che ha anno amo la una si fai sono di era

anno una si di che sono fai la per uomo era amo con non ha
con la non per fai era anno di amo ha si che uomo una sono
che uomo la sono si per con non una fai anno di era ha amo
di si era anno una amo uomo ha che sono per non fai la con
fai amo con ha non di che si era la una per anno sono uomo

una che sono fai uomo anno amo per ha di la con non era si
non anno per era con la sono una fai si uomo ha amo di che
sono era di uomo per si la anno con amo non fai ha che una
amo con ha una era fai si che di non sono anno la uomo per
la fai anno amo sono non una era uomo per ha si che con di

una anno che con si uomo sono amo per di la ha era non fai
era non uomo di amo la con che una ha anno fai sono per si
amo fai la sono per una anno si non era ha di uomo che con
sono si con era non che ha uomo fai la per amo una anno di
fai per una uomo anno di non era sono amo si che con ha la

di uomo era non la amo si ha che anno fai sono per con una
che la ha per una sono fai anno uomo non con era di si amo
anno una si che di con era non la per amo uomo ha fai sono
uomo sono di ha fai non la una amo si che con anno era per
con era fai si uomo ha per di anno sono non una la amo che

che con uomo anno sono si amo per era di ha non la fai una
era una non fai che la con anno uomo sono si per amo di ha
la ha sono di era uomo una si amo non anno fai che con per
non amo per la ha anno di che fai una era sono si uomo con
fai si che una con per sono la ha anno uomo amo non era di

uomo anno amo non di ha che era la fai con si per una sono
di sono con uomo amo era anno fai per si che una ha la non
con non era sono per una ha uomo che amo di la anno si fai
per che la ha fai non era di si con sono anno una amo uomo
una fai di si la amo uomo sono anno per non era con ha che

uomo sono che la con per fai una anno non di amo ha si era
fai di ha per si uomo la che amo con era una anno sono non
si una con anno era amo non ha sono per fai la che di uomo
di era non amo una sono anno la fai ha che uomo si per con
sono amo la ha non di che era uomo una anno per con fai si

la che si uomo di ha per amo con fai sono era non una anno
ha fai per si la con una uomo non che amo anno di era sono
per con una non anno fai sono di ha era la si uomo che amo
una anno uomo sono per era con non che di si fai amo la ha
era uomo amo fai che anno di con una si per ha sono non la

Supplementary material

Supplementary material to the paper

"Short Italian Wilkins Rate of Reading Test for repeated-measures designs in optometry and neuropsychology"

Maria De Luca[1†], Davide Nardo[2†], Giulia Carlotta Rizzo[3,4*], Roberta Daini[4,5], Silvia Tavazzi[3,4], and Fabrizio Zeri[3,4]

[1] IRCCS Fondazione Santa Lucia, Rome, Italy

[2] Department of Education, University of Roma Tre, Rome, Italy

[3] Department of Materials Science, University of Milano-Bicocca, Milan, Italy

[4] COMiB Research Centre in Optics and Optometry, University of Milano-Bicocca, Milan, Italy

[5] Department of Psychology, University of Milano-Bicocca, Milan, Italy

[†] These authors contributed equally to this work and share first authorship

[*] corresponding author

De Luca et al. (2024) Short Italian Wilkins Rate of Reading Test for repeated-measures designs in optometry and neuropsychology. *Front. Psychol.* 15:1448817. doi: 10.3389/fpsyg.2024.1448817

1 Supplementary Materials and methods

1.1 Vision assessment

Participants underwent a preliminary comprehensive eye examination and vision assessment at the Research Centre in Optics and Optometry at the University of Milano-Bicocca (COMiB).

Ocular pathologies were assessed by direct ophthalmoscopy and slit-lamp biomicroscopy. Habitual optical correction was recorded for each participant. Non-cycloplegic subjective refraction was undertaken monocularly with a phoropter procedure, then a final binocular equalisation with dissociated testing was carried out to obtain the least minus/most plus correction compatible with good visual acuity. Non-cycloplegic subjective refraction achieved was reported in the spherocylindrical notation. For each eye, the Spherical Equivalent Refraction (SER) was calculated as the algebraic sum of the sphere and half of cylinder. High-contrast monocular best corrected visual acuity (BCVA) was measured at 5 m using Sloan letters displayed on an LCD optotype system (Vision Chart, CSO, Florence, Italy) in 5-letter lines with the letter size decreasing logarithmically according to the principle of the ETDRS chart (Ferris et al., 1982). The threshold (in logMAR units) was determined by a forced choice procedure and a letter-by-letter (0.02 logMAR) scoring criterion. The letters were generated at high contrast (97.8% ± 0.2), measured by a photometer (Chroma Meter CS-100A, Minolta, Osaka, Japan) under photopic conditions (450 ± 50 lux, measured by a luxmeter HT307, HT Italia, Faenza, Italy). Ocular motility was examined using an H pattern test, while the binocular amplitude of accommodation was measured by the Donders' push-up method using a Royal Air Force (RAF) rule (Bernell Corporation; Mishawaka, Indiana, USA) and N5 stimulus, moved at a constant rate of approximately 1 cm/s, starting from 30 cm (Burns et al., 2020). The distance of the target representing both the first blur and total blur was recorded. The binocular accommodation facility at 40 cm, with ±2.00 diopters lenses, was tested for 1 min. The number of cycles in 1 min (one cycle consists of clearing a 6/9 target both with the plus and minus lenses) was recorded (Elliott, 2008). The near point of convergence test was performed by slowly moving an accommodative target (a single letter of approximately 0.2 logMAR equivalent at 40 cm) towards the bridge of the patient's nose, until the subject either reported diplopia or the examiner noticed a break in fusion. The distance to recover binocular fusion was also recorded. Stereoacuity was measured at 40 cm with the Wirt circles of the FLY Stereo Acuity Test (Vision Assessment Corporation; Elk Grove Village, United States).

2 Supplementary Results

2.1 Vision assessment

The group average outcomes are reported in **Table S1**. There were 29 participants already wearing an optical correction (spectacles or contact lenses). For these 29 participants, the subjective refraction measured during the optometric assessment showed negligible differences with the habitual correction: -0.04 ± 0.26 diopters (range -0.75 – 0.63) and -0.01 ± 0.28 diopters (range -0.50 – 0.88) in the right eye and left eye, respectively. This difference was considered unlikely to interfere with the reading task, therefore these participants retained their refractive correction (spectacles or contact lenses) during the reading sessions (both Radner Reading Charts and SI-WRRT), as normally used for reading and studying. Among the remaining 11 participants who did not wear any optical correction before being enrolled in the study, the subjective refraction measured during the optometric assessment showed negligible differences with respect to the absence of any optical correction (namely 0.00 diopters): 0.02 ± 0.39 diopters (range -0.75

– 0.63) and 0.05 ± 0.35 diopters (range -0.75 – 0.50) in the right eye and left eye, respectively. This difference was considered unlikely to interfere with reading. Therefore these participants performed the reading tasks (both Radner Reading Charts and SI-WRRT), without any optical correction.

TABLE S1 Optometric characteristics of participants. Legend: RE, right eye; LE, left eye; SER, spherical equivalent refraction; D, dioptres; BCVA, best corrected visual acuity; logMAR, logarithm of the minimum angle of resolution; NPC, near point of convergence; arcsec, arc seconds; SD, standard deviation.

Parameter	Mean	Median	SD	Min	Max
RE SER (D) of subjective refraction	-1.76	-0.88	2.37	-9.63	2.25
LE SER (D) of subjective refraction	-1.75	-1.13	2.43	-10.13	2.00
RE BCVA (logMAR)	-0.14	-0.14	0.05	-0.24	-0.02
LE BCVA (logMAR)	-0.14	-0.16	0.06	-0.24	-0.02
NPC Break (cm)	5.5	6.0	3.3	0.0	11.5
NPC Recovery (cm)	8.0	8.5	4.5	0.0	15.5
Binocular amplitude of accommodation -first blur (D)	10.6	9.8	3.1	6.5	20.0
Binocular amplitude of accommodation -total blur (D)	16.8	16.7	4.7	9.1	25.0
Accommodation facility (cycles per min)	12.1	12.5	4.4	4.5	19.0
Stereoacuity (arcsec)	23.1	20.0	4.4	20.0	32.0

Supplementary References

Burns, D. H., Allen, P. M., Edgar, D. F., and Evans, B. J. W. (2020). Sources of error in clinical measurement of the amplitude of accommodation. *J. Optom*. 13(1), 3–14. doi: 10.1016/j.optom.2019.05.002.

Elliott, D. B. (2013). Clinical procedures in primary eye care. (Fourth Edition). Saunders Ltd.

Ferris, F. L. 3rd, Kassoff, A., Bresnick, G. H., and Bailey, I. (1982). New visual acuity charts for clinical research. *Am. J. Ophthalmol*. 94(1), 91–96. PMID: 7091289.
